# Does Fever Response to Acetaminophen Predict Bloodstream Infections in Febrile Neutropenia?

**DOI:** 10.7759/cureus.36712

**Published:** 2023-03-26

**Authors:** Duncan B Mackie, Dennis Kuo, Megan Paul, Jennifer Elster

**Affiliations:** 1 Pediatrics, Ann & Robert H. Lurie Children's Hospital of Chicago, Chicago, USA; 2 Pediatric Hematology/Oncology, University of California San Diego, La Jolla, USA

**Keywords:** blood stream infections, pediatric oncology, temperature response, febrile neutropenia, bacteremia

## Abstract

Background: There is a need to identify clinical parameters for early and effective risk stratification and prediction of bacterial bloodstream infections (BSIs) in patients with febrile neutropenia (FN). Acetaminophen is used widely to treat fever in FN; however, little research exists on whether fever response to acetaminophen can be used as a predictor of BSIs.

Objectives: Investigate the relationship between fever response to acetaminophen and bacteremia in FN.

Design/Method: A retrospective review of patients (1-21 years old) presenting with FN and bacteremia at Rady Children’s Hospital (2012-2018) was performed. Demographic information, presenting signs/symptoms, degree of neutropenia (absolute neutrophil count (ANC) > 500 or < 500 cells/µL), absolute monocyte count, blood culture results, temperatures one, two, and six hours after acetaminophen, and timing of antibiotic administration were examined. Patients were stratified into three malignancy categories: leukemia/lymphoma, solid tumor, and hematopoietic stem cell transplant. Patients were matched with culture-negative controls based on sex, age, malignancy category, and degree of neutropenia.

Results: Thirty-five case-control pairs met inclusion criteria (70 presentations of FN). The mean age of the cases was 10.7 years (± 6.3) vs. 10.0 years (± 5.9) for the controls. Twenty were female (57%). Twenty-three pairs were categorized as leukemia/lymphoma (66%), eight as solid tumors (23%), and four as HSCT (11%). Thirty-four pairs (97%) had a presenting ANC < 500 cells/µL. Higher temperature one-hour post-acetaminophen was associated with bacteremia (p = 0.04). Logistic regression demonstrated that temperature one-hour post-acetaminophen had a significant predictive value for bacteremia (p = 0.011). The area under the receiver operating characteristic curves for logistic regression and classification and regression tree analysis were 0.70 and 0.71, respectively.

Conclusion: While temperature one-hour post-acetaminophen was higher among patients with bacteremia and was a significant predictor of bacteremia, fever response in isolation lacks sufficient predictive value to impact clinical decision-making. Future studies are needed to assess fever responsiveness as an adjunct to existing modalities of FN risk stratification.

## Introduction

Febrile neutropenia (FN) is a common complication of chemotherapy in the setting of pediatric malignancy and is the leading cause of emergency hospitalization among these patients [1,2]. As many as one-third of pediatric oncology patients receiving chemotherapy or hematopoietic stem cell transplantation (HSCT) experience at least one instance of FN, with similar rates of FN in both leukemic and solid tumor disease [2,3]. While most FN episodes are not associated with significant bacterial infection, bacterial bloodstream infections (BSIs) are the leading infectious cause of FN and thus account for significant morbidity and mortality [2,4]. Given the mortality associated with BSIs, the current pediatric FN standard of care calls for inpatient observation and empiric broad-spectrum antibiotics. This decreases mortality from BSIs but also results in hospitalization and antibiotic therapy for many patients with more benign causes of their FN. For this reason, substantial efforts have been made to establish and validate reliable clinical decision rules (CDRs) to predict bacteremia and severe bacterial infections (SBIs) in neutropenic patients. These CDRs most commonly utilize a combination of patient-/disease-related factors, presenting signs/symptoms, and results of laboratory tests and biomarkers [5-11]. Despite these efforts, no singular CDR has emerged as a superior method for risk stratification with current recommendations suggesting that pediatric centers adopt CDRs on an individual basis considering institutional resources and local validation [1].

Acetaminophen is widely utilized for symptom control in patients with FN due to its analgesic and antipyretic effects. It has been shown to significantly lower peak temperatures in FN compared to placebo and is a first-line agent for temperature control in FN [12-14]. Many CDRs utilize peak temperatures or set temperature cutoffs (e.g., ≥ 39.0°C) in an attempt to gauge the likelihood of bacteremia or severe bacterial infection in FN [5,15-18]. Despite the widespread use of acetaminophen in FN and the utilization of temperature for FN risk stratification, the efficacy of fever responsiveness to acetaminophen as a means of risk stratification or a predictor of bacteremia has not yet been determined.

## Materials and methods

Setting and study population

This is a single-center retrospective review of pediatric cancer patients presenting with FN and culture-confirmed bacteremia at Rady Children’s Hospital between 2012 and 2018. The study was approved by the Institutional Review Board. Patients >1 year old and <21 years old with a documented history of malignancy who presented to the emergency department with FN and bacteremia were included in the study. Patients were identified using the International Classification of Disease codes for FN and bacteremia. All identified electronic medical records underwent manual review, and patients were excluded if they did not have a documented malignancy, if they developed FN during hospital admission for another indication, if the review of clinical documentation indicated that blood culture positivity was due to a contaminant rather than true bloodstream infection, if they were treated at an outside facility prior to presentation, or if they received analgesic/antipyretic therapy with any non-steroidal anti-inflammatory drug medications in conjunction with acetaminophen. Study definitions used for fever, neutropenia, and bacteremia are detailed in Table 1.

**Table 1:** Study definitions ANC: absolute neutrophil count

Term	Definition
Fever	Documentation of a single temperature ≥38.0°C (100.4°F)
Neutropenia	ANC <1,500 cells/µL on presentation
Bacteremia	Presence of a bacterial pathogen on ≥1 blood culture set

Data collection

Demographic information, degree of neutropenia (absolute neutrophil count (ANC) <500 or >500 cells/µL), absolute monocyte count (AMC), presence of GI symptoms, chills/rigors, severe mucositis, hypotension, and evidence of focal infection were collected. Blood culture results, amount/timing of acetaminophen doses after hospital presentation, patient temperatures one, two, and six hours after acetaminophen, timing of antibiotic initiation, and presence of antibiotic prophylaxis prior to presentation were also examined. Blood culture data was collected upon presentation and prior to hospital admission. Temperature measurements within 30 minutes of the one, two, and six-hour time points were used for post-acetaminophen temperature documentation. Temperatures were collected using standard methods and a thermometer. Temperatures reported and antipyretic doses administered prior to hospital presentation were not included in the statistical analysis. The starting time point for each patient’s temperature analysis was defined as the first hospital-recorded temperature >38.0°C or 100.4°F. All acetaminophen doses for the cases and controls were administered enterally.

Patients were stratified into one of three malignancy categories: leukemia/lymphoma, solid tumor, and HSCT. All patients categorized as HSCT received a transplant for a primary malignancy. Each patient was paired with a matched control using sex, age, category of malignancy, and degree of neutropenia. Controls were identified as patients with documentation of FN and negative blood cultures during hospital admission. Controls additionally had no clinical documentation or test results consistent with a focal bacterial infection or a focal/disseminated fungal infection. Fifteen patients (43%) served as their own control using data from a separate culture-negative admission for FN. For the remaining 20 patients (57%), if multiple possible controls were identified, then the control with the same primary malignancy was used (e.g., case with AML and control with AML). In the event there were still multiple controls to choose from, then the control closest in age to the case was used. When using age as a variable for matching patients, all cases ≤5 years old were matched with controls ≤5 years old and vice versa. Prior to cohort assembly, a power analysis determined that data from 70 presentations of FN (35 cases and 35 controls) would be necessary to demonstrate the significance of a temperature change of 1°F or 0.56°C after acetaminophen administration at a power of 80%.

Statistical analysis

The categorical data is summarized as a number (percentage) and the continuous data is the mean (standard deviation). Significance was determined by the chi-square test (Fisher's exact test) for categorical variables and either Kruskal-Wallis (non-parametric) or one-way ANOVA (parametric) testing for continuous variables. Normality for all continuous variables was determined using skewness and kurtosis. Initial regression analysis and receiver operating characteristic (ROC) curve generation for predictors of blood culture positivity were performed using multivariate stepwise binary logistic regression (ɑ to enter model = 0.05 and ɑ to exit model = 0.1). Machine learning via a classification and regression tree (CART) analysis was performed to model non-linear relationships between predictor variables of interest and bacteremia and to generate an optimal decision tree diagram. The CART model was first constructed using a training data set (70% of study population data), and then its efficacy was tested via a testing data set (30% of study population data). Statistical significance was defined as P ≤0.05. All statistical analysis and figures were obtained using Minitab® Statistical Software version 21.1.0 (Minitab, State College, Pennsylvania).

## Results

A total of 35 patients presenting with FN and culture-confirmed bacteremia and 35 matched controls (sex, age, category of malignancy, and degree of neutropenia) with negative blood cultures were included in the study (70 total presentations of FN). Beyond having negative blood cultures, controls did not demonstrate diagnostic testing (serologic testing, tissue cultures, Karius testing, etc.) concerning non-hematogenous bacterial or fungal infections. Nine of the 35 controls (26%) had a positive respiratory viral panel which was considered to be the cause of their fever, while the remaining 26 (74%) were considered to have FN of an unknown cause. None of the patients identified as cases had documented fungemia or evidence of focal fungal infection. All controls experienced spontaneous resolution of symptoms with symptomatic treatment and were discharged home without complications (development of healthcare-associated infection, sepsis, need for ICU admission, or death).

Patient demographic data and the frequency of presenting signs/symptoms often used for FN risk stratification are included in Table 2.

**Table 2:** Patient characteristics and presenting signs/symptoms ϯ = mean (± standard deviation); HSCT = hematopoietic stem cell transplantation, ANC = absolute neutrophil count, AMC = absolute monocyte count, GI symptoms = vomiting/diarrhea/constipation or abdominal pain

Characteristic	Cases (n = 35)	Controls (n = 35)	p-value
Age at presentation (years)	10.7 (±6.3)^ϯ^	10.0 (±5.9)^ϯ^	1.0
Gender (female)	20 (57%)	20 (57%)	1.0
Malignancy category			1.0
Leukemia/lymphoma	23 (66%)	23 (66%)	
Solid tumor	8 (23%)	8 (23%)	
HSCT	4 (11%)	4 (11%)	
ANC on presentation (cells/µL)			1.0
<500	34 (97%)	34 (97%)	
≥500	1 (3%)	1 (3%)	
AMC on presentation (cells/µL)			1.0
<100	22 (63%)	24 (69%)	
≥100	8 (23%)	8 (23%)	
Data missing	5 (14%)	3 (9%)	
GI symptoms on presentation			0.33
Present	11 (31%)	16 (46%)	
Absent	24 (69%)	19 (54%)	
Chills/rigors on presentation			0.43
Present	5 (14%)	2 (6%)	
Absent	30 (86%)	33 (94%)	
Severe mucositis			1.0
Present	2 (%)	1 (3%)	
Absent	33 (%)	34 (97%)	
Hypotension			0.01
Present	7 (20%)	0 (0%)	
Absent	28 (80%)	35 (100%)	
Evidence of focal infection			0.01
Present	7 (20%)	0 (0%)	
Absent	28 (80%)	35 (100%)	

Overall age ranged between 1.4 and 18.8 years old. The average age was 10.7 (±6.3) and 10.0 (±5.9) years for the cases and controls respectively. Twenty patients were female (57%), and 34 (97%) had presenting ANCs <500 cells/µL. AMC was < 100 cells/µL in 24 of the controls (69%) and 22 (63%) of the cases. Twenty-three matched case-control patient pairs (66%) were categorized as having leukemia or lymphoma, eight (23%) as having a solid tumor, and four (11%) as being status-post HSCT.

The presence of a pre-acetaminophen temperature ≥39°C was significantly more common among culture-positive patients (p = 0.044); however, there was no statistically significant difference in the max temperature observed (Tmax) between the two groups (p = 0.080). Temperatures one hour after acetaminophen administration were significantly different between the bacteremic positive cases and negative controls (p = 0.040). These cases were also found to have a higher mean temperature one hour after acetaminophen administration (101.2°F (SD ±1.87, range: 97.9 - 106.3) vs. 100.0°F (SD ±1.60, range: 97.5 - 104.3)) for the controls. Pre-acetaminophen temperature and the total number of days of fever were not significantly different between the groups (p = 0.71 and 0.20, respectively).

Despite these distinctions, no significant relationship was seen between the presence of any fever (as defined by a temperature ≥100.4°F/38.0°C) one hour after acetaminophen and bacteremia (p = 0.16). Temperatures two and six hours after acetaminophen administration were not significantly different between the groups (p = 0.35 and 0.15, respectively). The absolute value of changes in patient temperatures between the pre-acetaminophen, one-, two-, and six-hour time points were also not significantly different between the groups. The mean mg/kg dosing of acetaminophen was not significantly different between groups (p = 0.35). Details regarding average values and significance of different temperature endpoints are detailed in Table 3.

**Table 3:** Pre- and post-acetaminophen temperature data Tmax = maximum recorded temperature during hospitalization; STD = standard deviation

Characteristic	Cases (n = 35)	Controls (n = 35)	p-value
Tmax (mean ±STD)	103.1°F/39.5°C (±1.6/0.89)	102.3°F/39.1°C (± 1.3/0.74)	0.080
Total days of fever	2.3 (±1.8)	2.3 (±2.2)	0.73
Pre-acetaminophen temperature (mean ±STD)	101.7°F/38.7°C (±1.2/0.65)	101.4°F/38.6°C (±1.2/0.66)	0.20
Pre-acetaminophen temperature > 39 °C	12 (34%)	4 (11%)	0.044
Temp. one-hour post-acetaminophen (mean ±STD)	101.2°F/38.4°C (±1.9/1.0)	100.0°F/37.8°C (±1.6/0.89)	0.040
Temp. two-hour post-acetaminophen (mean ±STD)	100.5°F/38.1°C (±1.9/1.1)	99.1°F/37.3°C (±1.3/0.73)	0.35
Temp. six-hour post-acetaminophen (mean ±STD)	99.3°F/°C (±1.7/0.97)	99.2°F/°C (±1.5/0.82)	0.15

Data regarding the timing of antibiotic administration was collected for both groups to account for possible confounding impacts of early antibiotic administration on temperature among patients with bacteremia. Analysis showed no difference between the groups in the duration of antibiotic coverage (hours) at the one-hour post-acetaminophen time point (p = 0.14). Additionally, linear regression analysis demonstrated that the duration of antibiotic coverage (hours) at the one-hour post-acetaminophen time point was not predictive of patient temperatures among those patients with positive blood cultures (p = 0.22). Two of the cases were on prophylactic levofloxacin at the time of presentation; however, administration of prophylactic levofloxacin was not found to differ significantly between cases and controls (p = 0.49). There was also no significant relationship found between the cause of bacteremia amongst the cases and their corresponding temperature one-hour post-acetaminophen administration (p = 0.78).

**Figure 1:**
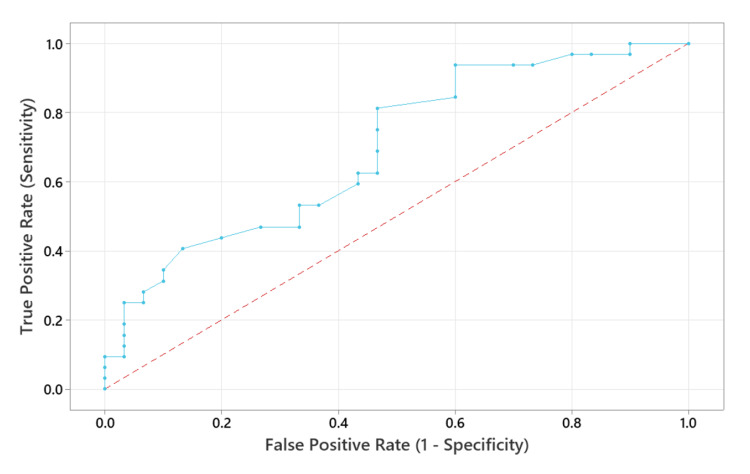
Binary logistic regression ROC curve ROC curve constructed from binary logistic regression analyzing temperature one-hour post-acetaminophen’s ability to predict bacteremia among this patient population. AUC = 0.70.

Given the significant relationship seen between patients with culture-confirmed bacteremia and temperatures one-hour after initial acetaminophen administration, pre-acetaminophen temperature ≥39°C, evidence of focal infection and hypotension, a multivariate logistic regression was performed to assess the ability of these variables to predict bacteremia in this patient population. Regression modeling demonstrated that temperature one hour after acetaminophen was the only variable with a significant predictive value for bacteremia (p = 0.011). A ROC curve was generated using this regression model (Figure 1) and demonstrated an area under the curve (AUC) of 0.70.

**Figure 2:**
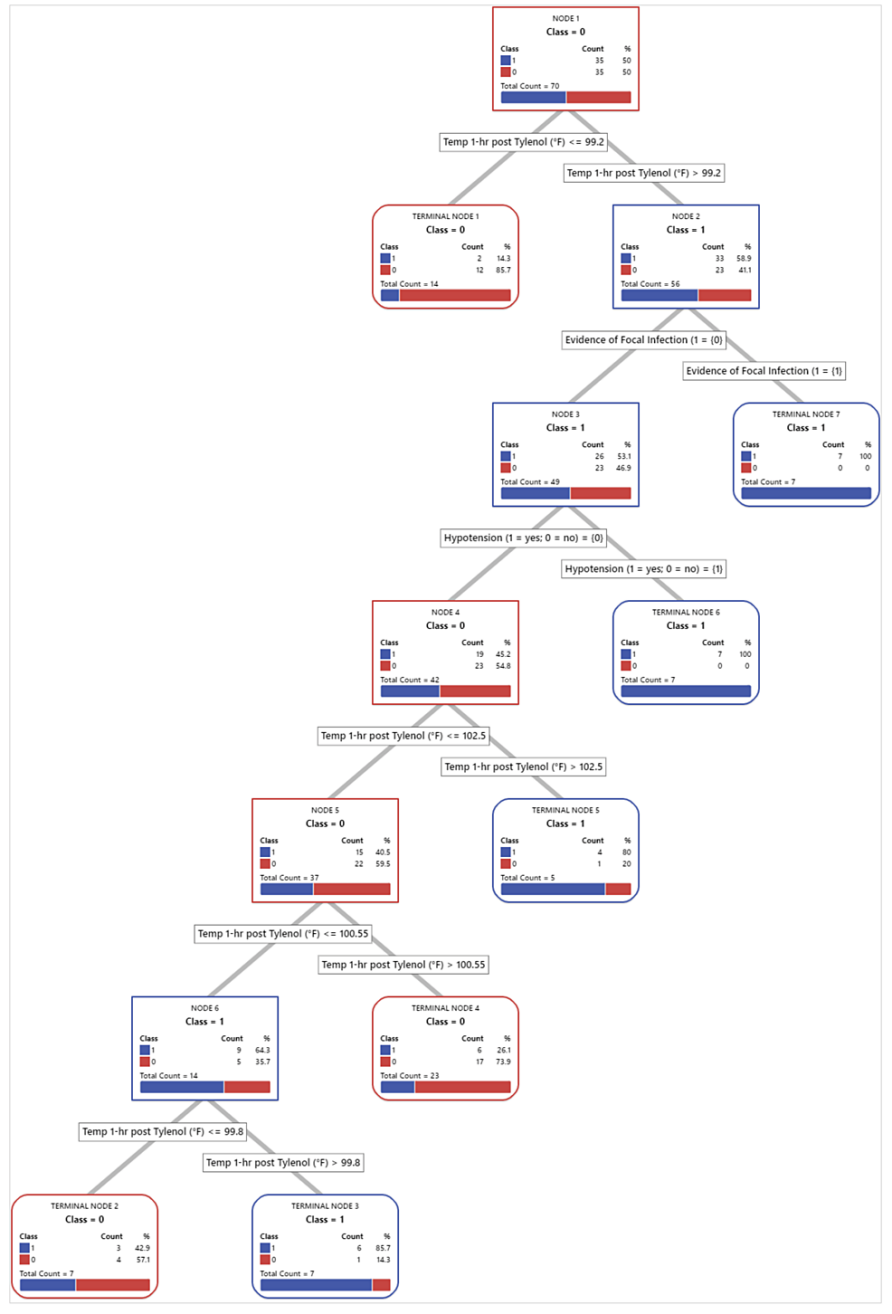
CART optimal decision tree diagram CART analysis utilizing temperature one hour after acetaminophen, presence of pre-acetaminophen temperature ≥39.0°C, evidence of focal infection, and hypotension to generate an optimal tree diagram for determination of bacteremia. Class 1 = cases (bacteremia). Class 2 = controls (blood culture negative).

CART analysis was also performed to construct an additional model and identify optimal breakpoints in temperature one hour after acetaminophen administration. Modeling was again performed with the inclusion of pre-acetaminophen temperature ≥39°C, evidence of focal infection, and hypotension variables. An optimal decision tree for the determination of bacteremia was constructed and is detailed in Figure 2.

**Figure 3:**
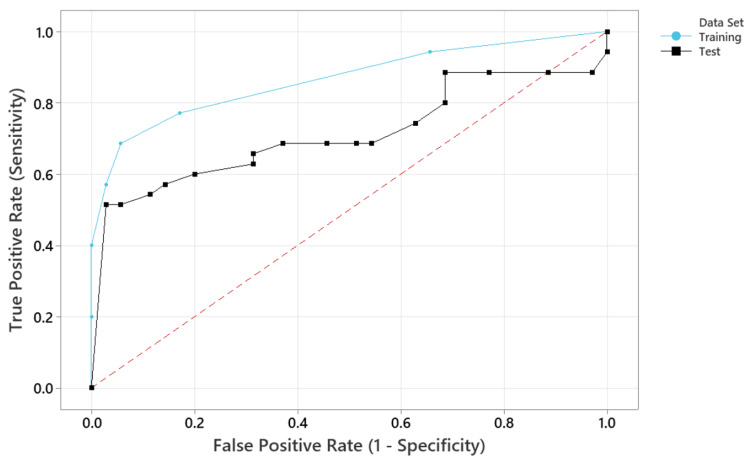
CART ROC curve ROC curve constructed from CART analysis. Patient data was split into training (70%) and testing arms (30%) for machine learning model construction and validation. AUC for training data set was 0.86 vs 0.71 for the test set.

Sensitivity and specificity for the CART model were 62.9% and 74.3%, respectively. The AUC-ROC of the CART model for the test arm was 0.71 (Figure 3).

**Figure 4:**
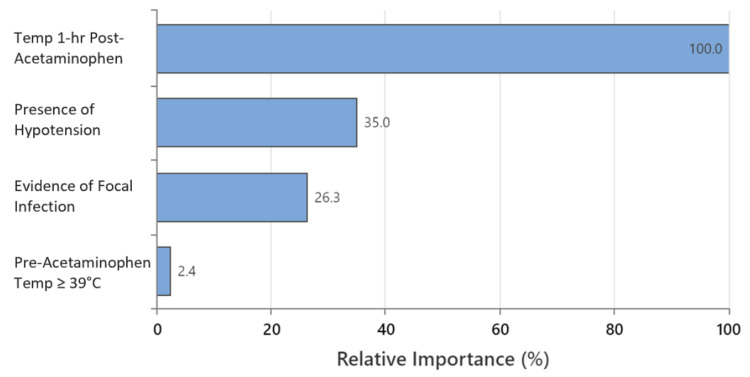
Relative variable importance Relative variable importance for the optimal tree diagram generated by characterization and regression tree analysis. Variable importance measures model improvement when splits are made on a predictor in the optimal tree diagram. Relative importance is defined as % improvement with respect to the top predictor.

Examination of the CART decision tree breakpoints demonstrated that 12 of 14 patients (85.7%) with temperature ≤99.2°F/37.3°C one hour after acetaminophen were blood culture negative. Likewise, four of five patients (80%) with temperatures >102.5°F/39.2°C one hour after acetaminophen were culture positive. All patients with hypotension or evidence of focal infection displayed positive blood culture results. Relative variable importance analysis (defined as % improvement with respect to the CART model’s top predictor) demonstrated that temperature one hour after acetaminophen was the model’s most important variable for predicting bacteremia (Figure 4).

## Discussion

This study identified that temperature one hour after initial acetaminophen dosing was significantly higher in individuals with culture-confirmed bacteremia. We demonstrated that temperature one hour after initial acetaminophen had significant value in predicting bacteremia using both logistic regression and a CART machine learning model, with AUC-ROC comparable to other prospectively validated CDRs [16,19-22]. Similar to prior reports, we observed that presenting temperature ≥39.0°C, evidence of focal infection, and hypotension were also all predictive of bacteremia [5,8,9,15]. However, when examining the relative importance of input variables in our CART model, temperature one hour after acetaminophen was by far the most significant predictor of bacteremia when compared to pre-acetaminophen temperature ≥39.0°C, evidence of focal infection, and hypotension. Generation of an optimal decision tree using CART analysis also demonstrated that 85.7% of patients with temperature ≤37.3°C (99.2°F) one hour after acetaminophen had negative blood cultures. Conversely, 80% of patients with temperature >39.2°C (102.5°F) one hour after acetaminophen were culture positive. These results suggest there may be some utility in using post-acetaminophen temperatures at both extremes of the spectrum to aid current CDRs in identifying patients with low- and high-risk bacteremia. This may be especially useful in assessing patients with a mix of low- and high-risk features (e.g., high degree of neutropenia with a low-risk category of malignancy), in which prompt and accurate determination of risk can be difficult.

The temperature has primarily been used as a binary variable (e.g., temperature ≥ or <39.0°C) in CDRs for FN risk stratification, with few if any studies investigating the relationship between fever response to antipyretics and bacteremia. Alali et al. described the creation of a random forest model for predicting BSI and the need to transfer to an intensive care unit (TIC), in which the authors detail that treating temperature as a continuous variable and including peak temperature as an input variable increased model performance [16]. The authors also identified max temperature to be the most important predictor of bacteremia and the second most important predictor of TIC (behind only hypotension) [16]. While our study did not demonstrate a significant difference in Tmax between patients with bacteremia and culture-negative individuals, the concept of using temperature to predict bacteremia, as well as the relative importance of temperature as a predictor of bacteremia, is congruent with the results of this study.

Another study by Haeusler et al. utilized prospective data from the Australian predicting infectious complications in children with cancer study to investigate the association between 10 commonly utilized CDR variables and the presence of bacterial infection [22]. Using both univariate and multivariate analysis of 858 episodes of FN, the authors demonstrated that increasing temperature was significantly associated with bacterial infection [22] and was one of only three variables that maintained significant predictability for bacteremia after multivariate analysis (the other two being decreasing platelet count and appearing clinically unwell) [22]. These results again highlight the important relationship between temperature and bacteremia in FN and lend credence to the idea that fever response one hour after acetaminophen can lend valuable insight into which patients are likely to have bacteremia and should not be categorized as low risk.

Many contemporary CDRs utilize a host of patient or disease-related factors to help risk stratify patients presenting with FN (a specific type of leukemia/lymphoma, presence of relapse with marrow involvement, chemotherapy within seven days of FN presentation, etc.) [6-8,10]. To control these factors, we split patients into groups based on their category of malignancy (leukemia/lymphoma, solid tumor, or HSCT) and matched them based not only on this category but also on the degree of neutropenia, using this as a proxy for the degree of immunosuppression. Another commonly utilized patient factor for risk stratification is the presence of a central venous catheter (CVC). CVCs are present almost ubiquitously across this institution for patients undergoing active treatment, and thus their presence was deemed largely unhelpful and non-discriminatory for the purposes of this investigation. Rondinelli et al. also cited age as a patient factor that could increase the risk of serious infectious complications, assigning increased risk to patients ≤5 years old. In our study, by matching cases-controls based on age and ensuring all cases ≤5 years old were also matched with controls ≤5 years old, we made the response to the acetaminophen variable independent of age.

Several laboratory results and biomarkers are also commonly utilized in FN CDRs. Inflammatory markers such as C-reactive protein (CRP), procalcitonin (PCT), interleukin-6 (IL-6), or interleukin-8 (IL-8), have all been touted as helpful means of FN risk stratification [6,10,23-25]. The use of CRP, PCT, IL-6, and IL-8 as an adjunct to fever response to acetaminophen was investigated for this study; however, at our institution like many others, it is not routine practice to obtain these inflammatory markers on presentation and thus the retrospective data available for our patient population was too sparse to provide any meaningful insights. AMC is another laboratory variable seen in several CDRs. AMC cutoffs for the determination of high-risk patients range between <100 cells/µL to <155 cells/µL depending on the CDR [5,11,26]. In this study, we did not see a significant relationship between AMC and bacteremia at a cutoff of <100 cells/µL. One prospective validation study of several CDRs found that by altering the cutoff parameters and recalibrating several of these CDRs, they were able to raise the overall AUC-ROC and low-risk yield for the risk stratification models [22]. These results were obtained in part by lowering the AMC cutoff from <100 or <155 cells/µL to <15 cells/µL, suggesting that the lack of significance between AMC and bacteremia in our study may be due in part to questions regarding the optimal AMC threshold.

Our study has several limitations. As an analysis of a single tertiary care academic medical center, our results may not necessarily be generalizable to other institutions. Our study is also limited by its retrospective nature that hindered the uniform collection and incorporation of clinical and laboratory variables (e.g., GI symptoms, chills/rigors, mucositis, evidence of focal infection, CRP, PCT, IL-6, IL-8) into our models. Similarly, the need to retrospectively collect specific post-acetaminophen temperature time points on patients and minimize possible confounding factors between the cases and controls (e.g., concomitant focal bacterial or fungal infection) limited inclusion of FN cases; however, all single-center retrospective analyses suffer similar drawbacks, making the need for prospective analysis and multicenter validation critical.

## Conclusions

In this study, we demonstrated that fever response one hour after administration of acetaminophen can be used to predict bacteremia among pediatric cancer patients presenting with FN. We modeled this using both binary logistic regression and CART machine learning analysis with AUC-ROC of 0.70 and 0.71, respectively. While unlikely to provide sufficient predictive value to impact clinical decision-making in isolation, our results highlight a promising, objective, and novel means of prompt FN risk stratification that has the potential to be used across a multitude of clinical settings. Future prospective, large cohort, multicenter studies are needed to assess the utility of fever responsiveness in the risk stratification in FN.
